# Identification of Branched and Linear Forms of PFOA and Potential Precursors: A User-Friendly SMILES Structure-based Approach

**DOI:** 10.3389/fenvs.2022.865488

**Published:** 2022-03-24

**Authors:** Ann M. Richard, Hannah Hidle, Grace Patlewicz, Antony J. Williams

**Affiliations:** 1 Center for Computational Toxicology & Exposure, Office of Research and Development, U.S. Environmental Protection Agency, Research Triangle Park, Triangle Park, NC, United States; 2 ORAU Student Services Contractor to Center for Computational Toxicology & Exposure, Office of Research and Development, U.S. Environmental Protection Agency, Research Triangle Park, Triangle Park, NC, United States

**Keywords:** PFOA, PFAS, SMILES, branching, cheminformatics

## Abstract

Perfluorooctanoic acid (PFOA) and related compounds are per- and polyfluorinated alkyl substances (PFASs) of concern from toxicological, environmental, and regulatory perspectives. In 2019, the Conference of the Parties to the Stockholm Convention on Persistent Organic Pollutants listed PFOA, its salts, and PFOA-related compounds in Annex A to the Convention. Additionally, the listing specifically included PFOA branched isomers and compounds containing a perfluoroheptyl (C7F15)C moiety, with some noted exclusions. A draft updated “Indicative List” of 393 PFASs (335 with defined structures), each specified as falling within or outside the listing, was released for comment in 2021. The U.S. Environmental Protection Agency’s CompTox Chemicals Dashboard has published a curated PFAS list containing more than 10,700 structures. Applying the PFOA and related compounds listing definition to screen this list required a structure-based approach capable of discerning salts and branched or linear forms of the (C7F15)C moiety. A PFOA SMILES workflow and associated Excel macro file, developed to address this need, applies a series of text substitution rules to a set of canonicalized SMILES structure representations to convert branched forms of the (C7F15)C moiety to linear forms to aid their detection. The approach correctly classified each Stockholm Convention draft Indicative List structure relative to the PFOA and related compounds definition, and accurately discerned branched and linear forms of the (C7F15)C moiety in over 10,700 PFAS structures with 100% sensitivity (no false negatives) and 99.7% accuracy (35 false positives). Approximately 20% of structures in the large PFAS list fell within the PFOA and related compounds definition, and 10% of those were branched. The present work highlights the need to computationally detect branched forms of PFASs and promotes the use of unambiguous, structure-based definitions, along with tools that are publicly available and easy to use, to support clear communication and regulatory action within the PFAS community.

## INTRODUCTION

### Background

Perfluorooctanoic acid (PFOA) and related compounds constitute a category of per- and polyfluorinated alkyl substances (PFASs) of ubiquitous occurrence and global concern from toxicological, environmental, and regulatory perspectives (U.S. EPA 2017; Sunderland et al., 2019). Despite the phasing out of production of PFOA over the past decade, high past production levels and widespread usage of PFOA in industrial processes and consumer products (such as carpeting, upholstery, apparel, and cookware, e.g., Teflon^©^) have left a significant environmental contamination legacy (Glüge et al., 2020). In 2019, the Conference of the Parties (COP) to the Stockholm Convention on Persistent Organic Pollutants (POP) decided to list PFOA, its salts, and PFOA-related compounds in Annex A to the Convention (Stockholm Convention POPRC 2019). More specifically, what we will henceforth refer to as the “SC PFOA listing” included PFOA, its salts and branched isomers, and compounds with the potential to degrade to PFOA, i.e., containing a perfluoroheptyl moiety with formula (C7F15)C. Some chemicals, such as perfluorooctanesulfonic acid (PFOS) and its salts, were excluded from the listing either due to their consideration elsewhere or due to their presumed inability to degrade to PFOA. The COP also invited Parties to provide further information regarding the identification of substances covered by the SC PFOA listing. The COP had previously requested that the Stockholm Convention Secretariat compile this information, in consultation with the Convention’s POP Review Committee, and establish an indicative list of compounds falling under the SC PFOA listing, make it available on the Convention’s website, and update it periodically (Stockholm Convention 2017). A draft updated PFOA “Indicative List” of 393 PFASs, each specified as falling within or outside of the SC PFOA listing, was released in 2021 for comment by the Stockholm Convention Parties and observers (Stockholm Convention POPRC 2021). This list, henceforth referred to as the “2021 PFOA Indicative List,” includes chemical names and Chemical Abstracts Registry Numbers (CASRNs) for each substance and serves as a useful reference table. However, a non-trivial challenge to the PFAS community is determining whether thousands of PFASs, not currently included on the 2021 PFOA Indicative List, fall within the SC PFOA listing description.

Buck et al. (2011) published an early attempt to generally define and apply standard naming conventions to common classes of PFASs. Today, there is no single community consensus definition of what constitutes a PFAS but the trend leans toward broadly inclusive definitions that cover small and large chemicals of potential concern, allowing for user-specific working scopes to be defined within these general parameters. Most recently, the Organisation for Cooperation and Economic Development (OECD) published endorsement of a broad PFAS definition as substances “that contain at least one fully fluorinated methyl or methylene carbon atom (without any H/Cl/Br/I),” i.e., requiring only a CF2 or CF3 group, with a few notable exceptions (Wang et al., 2021). For purposes of supporting programs within the U.S. Environmental Protection Agency (EPA), the CompTox Chemicals Dashboard (henceforth, referred to as “Dashboard”) has published a number of more constrained PFAS lists since March 2018. These have been bounded by specific substructural constraints that have been modified with successive list iterations to address the increasing breadth and complexity of the evolving PFAS structure space (Williams et al., 2022). The most recent list contains over 10,700 structures and is bounded by the presence of one of 6 PFAS substructural moieties (U.S. EPA PFASSTRUCTv4, 2021). This list includes more than 3700 curated PFAS structures extracted from the OECD Global PFAS List (2018), as well as curated structures from several other public lists associated with mass spectral libraries (e.g., U.S. EPA PFASTRIER, 2015; Norman Network, 2021) or compiled by regulatory bodies (e.g., U.S. EPA PFASKEMI, 2021; KEMI, 2015). To assess whether each of these structures falls within or outside of the SC PFOA listing, a computational, structure-based approach capable of detecting neutral and salt forms, perfluoro chain length, functional groups, and branched isomers of perfluoro chains is required. Furthermore, the approach should be publicly accessible and easy to apply to encourage use and adoption by the public, scientific and regulatory communities.

The explicit inclusion of branched isomers of PFOA and related compounds in the SC PFOA listing reflects a growing body of evidence indicating significant levels of occurrence of PFAS branched isomers in the environment and biota, as well as differential properties of linear versus branched isomers. The studies that exist relative to the issue of PFAS branched versus linear forms have primarily focused on PFOS and PFOA. As summarized in a recent review article by Shultz et al. (2020), the historical synthetic production of large quantities of PFOS and PFOA by electrochemical fluorination, largely replaced by telomerization in present day syntheses of PFASs, has led to significant environmental occurrence of branched isomers of PFOS (estimated at 20–30% of total occurrence) and somewhat less of PFOA (estimated at 15–20% of total occurrence). Branched isomers are more polar and hydrophilic, likely accounting for preferential occurrence of linear isomers in soil and branched isomers in water (Pellizzaro et al., 2018; Gao et al., 2019). Finally, there is evidence that linear versus branched isomers differ in their bioaccumulation properties (Beesoon and Martin 2015; Liu et al., 2018) and toxicity (Loveless et al., 2006; Liu et al., 2018; Wang et al., 2019).

In developing a structure-based approach for determining whether a substance falls within the SC PFOA listing, detection of branched isomers of a perfluoroalkyl chain of specified formula (e.g., C7F15) posed a surprisingly difficult cheminformatics challenge. Fragment-based approaches can, in principle, detect the presence of perfluoroalkyl chain branching but are unable to capture the concept of branching associated with an alkyl chain of specified formula without explicit inclusion of all branched possibilities. PFOA, for instance, has 38 unique branched isomers, whereas detection of a branched perfluoroheptyl (C7F15)C precursor embedded in larger molecules could yield even more possibilities. In the present study, we report development of a heuristic approach employing a commonly used, text-based structure representation consisting of a set of canonicalized SMILES, as implemented in the Dashboard, to convert branched (C7F15)C moieties to their corresponding linear form to aid in their detection. Each of the noted exceptions included in the SC PFOA listing is also amenable to detection and assignment within the SMILES-based approach. The approach employs a relatively simple series of SMILES substitution rules that have been implemented into an Excel VBA macro for public dissemination. We used the most recent 2021 PFOA Indicative List (335 structures) as a benchmark for initial validation of SMILES rules but added rules and extended the approach in application to an expanded list of more than 10,700 PFAS structures to account for the broader diversity of PFAS structures. These results not only demonstrate the feasibility of the SMILES-based approach to detect branched PFAS isomers but also provide a greatly expanded indicative list of compounds, labeled as to whether or not they fall within the SC PFOA listing, to support the international PFAS research and regulatory communities.

### Perfluorooctanoic Acid and Related Compounds Listing

For purposes of the present study objectives, it was important to parse the precise language in the Stockholm Convention SC PFOA listing. At the time of this writing, the SC PFOA document (Stockholm Convention POPRC 2019) lists the inclusive portion of the listing as covering:

“PFOA, including any of its branched isomers; its salts; and PFOA-related compounds which, for the purposes of this risk management evaluation, are any substances that degrade to PFOA, including any substances (including salts and polymers) having a linear or branched perfluoroheptyl group with the moiety (C7F15)C as one of the structural elements”.

Stated exclusions to the SC PFOA listing that are applicable to defined structures (i.e., excluding polymers or mixtures) are as follows:

“C8F17-X where X = F, Cl, Br; Perfluoroalkyl carboxylic and phosphonic acids (including their salts, esters, halides and anhydrides) with ≥8 perfluorinated carbons; Perfluoroalkane sulfonic acids (including their salts, esters, halides and anhydrides) with ≥9 perfluorinated carbons; and perfluorooctane sulfonic acid (PFOS), its salts and perfluorooctane sulfonyl fluoride (PFOSF). We will refer toa chemical that satisfies the SC PFOA listing as satisfying the “PFOA In-rule.” For present purposes, structures that satisfy the PFOA In-rule but meet one of the above exclusion criteria will be referred to as satisfying the “PFOA Out-rule”.

One additional substance explicitly excluded from the SC PFOA listing was listed in a Note to Table 2 of the 2021 PFOA Indicative list (N-EtFOSA, N-Ethyl-1,1,2,2,3,3,4,4,5,5,6,6,7,7,8,8,8-heptadecafluoro-1-octanesulfonamide, CAS No. 4151–50–2, listed as DTXSID1032646 on the Dashboard) (Stockholm Convention POPRC 2021). This chemical was added to the end of the 2021 PFOA Indicative List reproduced here and is included in the exceptions list for the present analysis.

### SMILES-Based Workflow

The difficulty of detecting branched isomers of PFOA or the associated perfluoroheptyl group moiety (C7F15)C using available substructural search methods, paired with the specialized software and expertise needed to apply such methods, led us to search for a more user-friendly means to achieve this goal. SMILES (Simplified Molecular-Input Line-Entry System) is a text-based structure representation technology developed in the early 1980s that is still in use today due to its human readability and wide adoption in chemistry software applications (Weininger 1988). For most computational chemistry database applications, however, SMILES have been largely replaced by the IUPAC InChI (International Chemical Identifier) text-based structure representations (Heller et al., 2015) which, unlike SMILES, are fully supported by publicly available software and, along with the associated hashed InChI-Key identifiers (27 characters in length), are designed to uniquely represent a chemical structure. The main disadvantage of InChI for present purposes is that it is not designed to be human readable, nor can structure fragments be discerned. A form of SMILES referred to as “canonical SMILES” has been implemented into most structure-handling software applications to enforce uniqueness of SMILES structure-representations within the application. However, due to a lack of standardized SMILES canonicalization rules across the community, SMILES consistency is rarely achieved across different applications.

EPA’s Dashboard is built upon EPA’s DSSTox substance database, which presently contains close to a million chemicals (Williams et al., 2017; Grulke et al., 2019). The DSSTox PFASSTRUCTv4 structure collection, currently exceeding 10,700 chemicals, is the largest collection of curated PFAS structures of any publicly available database to date. Canonicalized SMILES strings computed with the JChem cartridge in Marvin JS (v 17.26.0, ChemAxon, Boston, MA) are available for the entire DSSTox structure collection from the Dashboard and can be downloaded for any published PFAS list (such as PFASSTRUCTv4, PFASOECD, etc.). In addition, a user can download JChem SMILES for any set of DSSTox-registered chemicals through the Dashboard Batch search by inputting a list of chemical names, DTXSIDs, or CASRNs (Lowe and Williams 2021).

**FIGURE 1 |:**
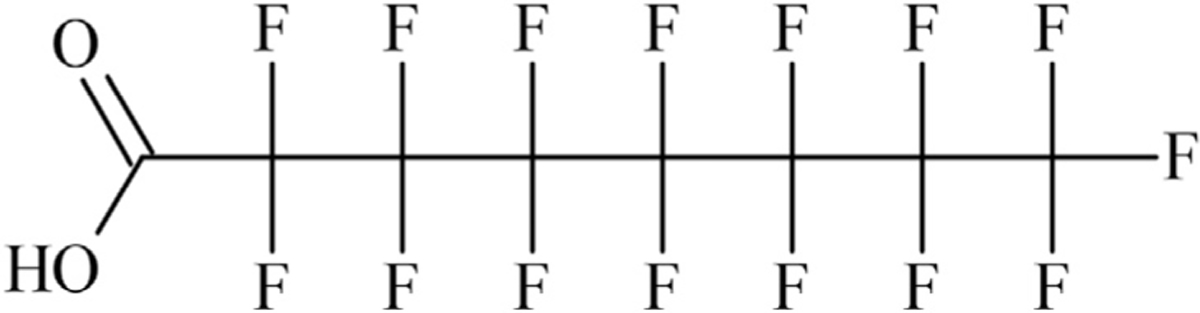
Linear form of perfluorooctanoic acid (PFOA).

The structure of the linear form of PFOA is shown in Figure 1.

This structure is represented by the following SMILES when the string is written starting from the terminal hydroxyl oxygen:

1. OC(=O)C(F)(F)C(F)(F)C(F)(F)C(F)(F)C(F)(F)C(F)(F) C(F)(F)(F)
  The following are just three of many possible alternative valid SMILES strings for PFOA, when the string is written starting at different atoms along the chain (note, hydrogens are usually implicit):
2. FC(F)(F)C(F)(F)C(F)(F)C(F)(F)C(F)(F)C(F)(F)C(F)(F)C(=O)O
3. FC(F)(C(F)(F)C(F)(F)C(F)(F)C(F)(F)(F))C(F)(F)C(F)(F)C(O)=O
4. FC(F)(C(F)(F)C(F)(F)C(O)(=O))C(F)(F)C(F)(F)C(F)(F)C(F)(F)(F)

The uniqueness issue with SMILES is addressed with canonicalization within an application (i.e., where only a single SMILES is generated for any chemical), but any one of the possible SMILES representations for PFOA listed above could be chosen by different canonicalization algorithms. Similarly, each of the 38 branched isomers of PFOA will present as many, if not more SMILES possibilities than for linear PFOA.

**FIGURE 2 |:**
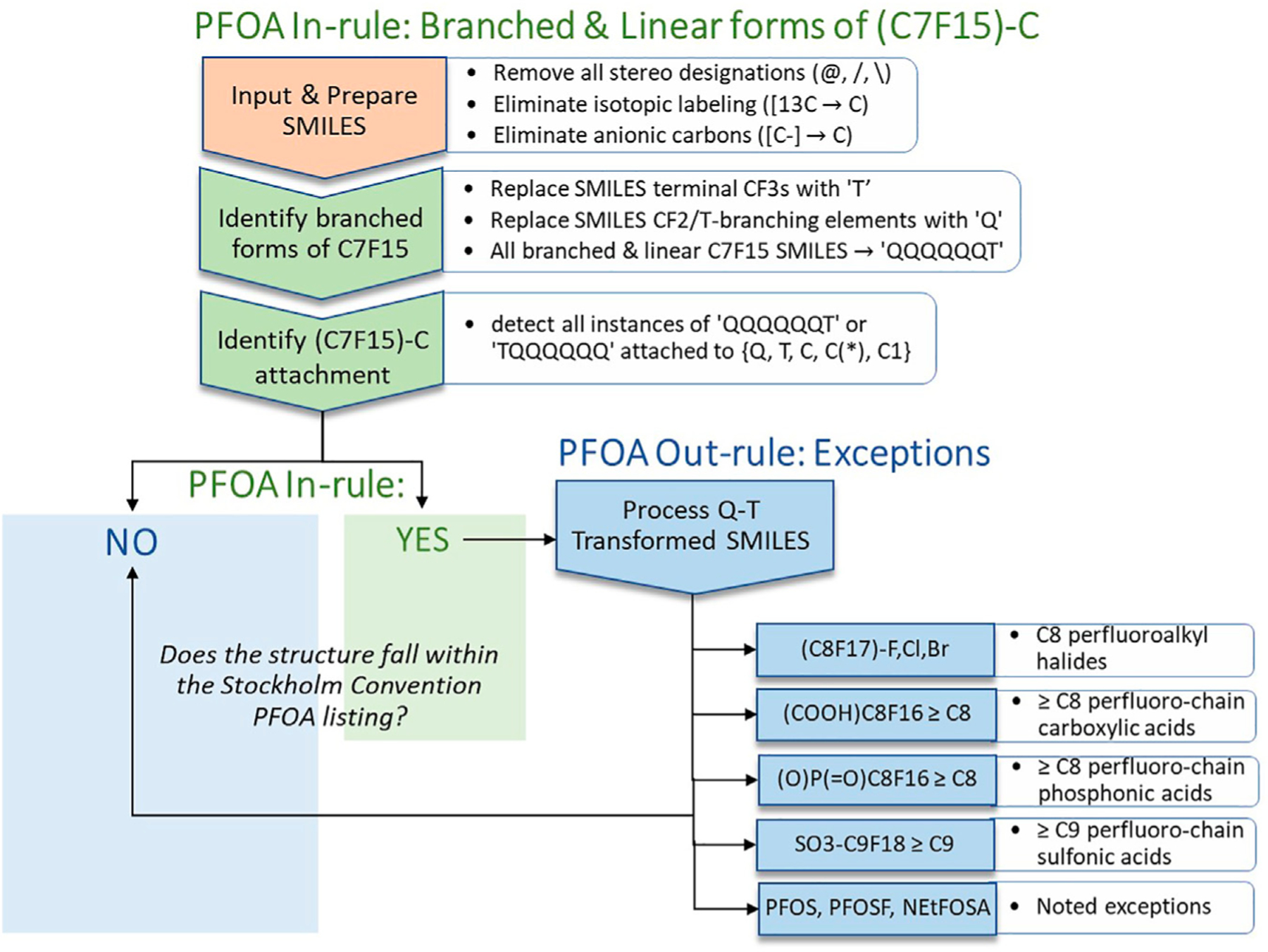
Schematic illustration of the SMILES-based implementation of the PFOA In-rule and PFOA Out-rule workflow steps leading to assignment of a structure (or list of structures) represented as SMILES (exported from EPA’s CompTox Chemicals Dashboard in JChem canonicalized format) as either YES (falling within the Stockholm Convention PFOA listing, with exclusions) or NO (not falling within the Stockholm Convention PFOA listing).

On examination of SMILES strings for the 2021 PFOA Indicative List exported from the Dashboard (generated by JChem), it was apparent that the perfluoroheptyl (C7F15)C moiety in each of the 38 branched PFOA isomers was bounded and contiguous within the SMILES strings (as in Options 1–3 above), i.e., the SMILES text representation was not split by the functional group as in Option 4. This enabled us to consider an approach involving simple SMILES text substitutions that would convert each of the 38 SMILES for branched isomers of PFOA to its corresponding linear form. Correctly identifying each instance of a (C7F15)C moiety in this subset and in the larger 2021 PFOA Indicative List of 335 structures provided a proof-of-concept of the approach. Extending the approach to a much larger, more structurally diverse PFAS set of over 10,000 chemicals posed a greater challenge but provided a more stringent validation of the approach and led to addition of some new rules and modifications. The portion of the final SMILES workflow pertaining to what we have termed the PFOA In-rule is shown in the upper section of Figure 2 below.

All structures that meet the conditions of the PFOA In-rule become candidates for falling within the SC PFOA listing. The lower right portion of Figure 2 identifies the PFOA Out-rule exceptions to the PFOA In-rule. A substance that passes through the PFOA In-rule as a “YES,” but satisfies one of the PFOA Out-rule exceptions, is labeled as a “NO,” i.e., as not falling within the SC PFOA listing.

## METHODS

### Mapping the 2021 Perfluorooctanoic Acid Indicative List to DSSTox Substances and Structures

The 2021 PFOA Indicative List Annex file was downloaded from the Stockholm Convention website (UNEP/POPS/POPRC.16 Follow-up, 2021) as a MS Word doc file. The document contains 2 tables that together constitute the Indicative List: Table 1 lists 351 PFASs designated as “covered by the listing of PFOA, its salts and PFOA-related compounds;” Table 2 lists 42 PFASs designated as “not covered by the listing of PFOA, its salts and PFOA-related compounds” (including the chemical explicitly mentioned in Table 2 Note). The two tables combined yielded a total of 393 PFASs, with CASRN, Category, Acronym(s), and Designation (chemical name) listed for the majority of entries. Included in the Category column in Table 1 were labels identifying each of the 38 PFOA branched isomers. We extracted the full PFOA Indicative List from the MS Word doc, combining the 2 tables into a single MS Excel table, and added a new column titled “2021 PFOA Indicative List Status” to indicate whether (YES - Table 1) or not (NO - Table 2) the substance was deemed to be covered by the SC PFOA listing. This combined 2021 PFOA Indicative List is available in Supplementary Table S1.

The combined listing of PFASs was then mapped to content in the DSSTox database based on either CASRN or chemical name to obtain the corresponding DSSTox substance mapping and associated chemical identifiers (DTXSID, DTXCID, Preferred Name, CASRN) and structure fields (e.g., formula, molecular weight, JChem SMILES, InChI). Any mappings that indicated a conflict in identifiers (e.g., names agreed, but CASRN did not) underwent additional manual curation review. In a small number of cases, the CASRN or substance name was not already registered in DSSTox, so a new DSSTox record was created. At the end of the review, all PFASs in the 2021PFOA Indicative List were mapped to DTXSID substance records (Supplementary Table S1). Of the 393 original entries, 335 were mapped to a record with a defined structure (i.e., DTXCID); the remaining 58 substances were determined to be polymers or mixture/formulations without a defined structure. A subset of the latter (30 total) were mapped in DSSTox to Markush-type structures, which provide a generalized structure representation that can be enumerated in the Dashboard to specific structurable members of a polymer or mixture family–see, e.g., https://comptox.epa.gov/dashboard/chemical/related-substances/DTXSID50897543 (toggle to view structures); these Markush structures were not, however considered further here. The final list of 335 structurable PFASs with their associated SMILES was moved forward in the SMILES workflow analysis.

### SMILES Inventory Lists

In addition to the 2021 PFOA Indicative List of 335 structurable PFASs, two additional JChem SMILES listings were compiled for inventory comparison and to process with the SMILES analysis workflow. PFASSTRUCTv4, previously mentioned, lists a total of 10,776 structures, each containing one or more of the 6 substructures defining the list. All but 10 of the PFASOECD list structures were contained within the PFASSTRUCTv4 list, and all but 8 of the 335 Indicative List structures were included in the other 2 lists. A file containing the combined join of the 3 inventory lists, separately indexed and totaling 10,794 structures, is provided in Supplementary Table S2 with the associated JChem SMILES and the “2021 PFOA Indicative List Status” column. This combined structure listing, henceforth referred to as “PFASSTRUCT+,” was processed through the initial SMILES Workflow and the results were used to iteratively improve and validate the final PFOA SMILES Workflow.

### Perfluorooctanoic Acid SMILES Workflow

An initial set of SMILES transformations for the In-rule and Out-rule workflows was judged sufficient when all 335 of the original 2021 PFOA Indicative List results were correctly predicted according to the original Table 1 (In-rule) and Table 2 (Out-rule) assignments. However, upon applying this initial set of SMILES transformations to the much larger PFASSTRUCT+ file, several transformations had to be modified or added to account for the significantly increased PFAS structural diversity of this larger set. To aid in the review and validation of the predicted assignments for this larger set of PFAS structures, we employed substructure search and structure viewing capabilities within the Spectrus/ChemSketch software (Advanced Chemistry Development, Inc., Toronto, Canada; v2017.2). The PFASSTRUCT+ DTXSID listing was used to query the DSSTox database and generate an SDF (structure data format) file. This file was then imported into the ACD/Labs software and substructure searches were performed to identify all structures containing linear forms of the C-C7F15 moiety, as well as structures containing linear forms of each of the Out-rule conditions listed above. All structure images generated for this report were also generated within the Spectrus software.

The complete list of SMILES transformations corresponding to the In-rule and Out-rule workflows, summarized in Figure 2, and referred to henceforth as the PFOA SMILES Workflow, are documented in Supplementary Tables S3, S4 along with intermediate and final predicted results columns for the PFASSTRUCT+ list, which includes the 335 structures on the 2021 PFOA Indicative List. Additionally, these In-rule and Out-rule SMILES transformations were incorporated into a VBA (Visual Basic for Applications) macro-enabled MS Office Excel file (Microsoft 365) (Suppl. PFOA_SMILES_macro_v1. xlsm file). The macro-enabled Excel file is set up to process an input data column of SMILES, either downloaded from the Dashboard or generated elsewhere using JChem, and produce a column of “YES” (In-rule) or “NO” (Out-rule) results to indicate whether the SMILES structure is predicted to fall within or outside of the SC PFOA listing, respectively. The macro-enabled file generates detailed results of the In-rule and Out-rule sections of the workflow to enable a user to identify all potential candidates for the In-rule, as well as to see when the Out-rule exclusions serve to negate some In-rule predictions from further consideration. To generate results using the macro (xlsm) file, a user need only enable the MS Excel macro functionality within the file, paste a column of JChem SMILES to a new worksheet, and run the macro; detailed instructions are provided in the Suppl. PFOA_SMILES_macro_v1. xlsm file.

## RESULTS

Application of the final PFOA SMILES Workflow to the original 2021 PFOA Indicative List of 335 PFAS chemicals for which structures were available yielded results that predicted with 100% accuracy each of the manual assignments from the original source document (UNEP/POPS/POPRC.16 Follow-up, 2021), i.e., the 299 structures falling within the conditions of the SC PFOA listing (YES) and the 36 structures not falling within the listing (NO). These, and the results obtained when the final PFOA SMILES Workflow was applied to processing of the full set of 10,794 SMILES in the combined PFASSTRUCT+ file, are summarized in Table 1.

Note that the use of term “model” has been avoided here due to the largely heuristic nature of our approach, which involved iterative SMILES transformations to reproduce the desired outcome, i.e., the “model” was fit to the dataset in hand. In the case of the 2021 PFOA Indicative List, published assignments (i.e., in or out of the PFOA Definition) were manually reviewed to confirm their accuracy. In the case of the much larger PFASSTRUCT+ file, a combination of substructure searching for linear forms of the perfluoroheptyl chain, which constituted the majority of cases, was combined with manual review of the remaining “YES” predictions, presumed to be branched by default. Visual inspection of the latter subset identified a total of 36 incorrect In-rule YES predictions, i.e., 36 False Positives, one of which satisfied an Out-rule, so was reversed to the correct “NO” prediction.

A similar combination of substructure searching with manual review of cases meeting the exception criteria for the SC PFOA listing was used to review the accuracy of the negative In-rule and Out-rule predictions. To confirm the lack of (C7F15)C branched forms within the larger set of 8461 negative predictions from the In-rule, we first filtered out any structures with formulae having fewer than 8 carbons or 15 fluorines, and then did a substructure search for the joint presence of two fragments required for a branched C7F15 moiety: CF3-C(C,F)(C,F), where (C,F) indicates either a C or F attachment, and a second CF3. The resulting set of approximately 500 structures was manually scanned for presence of (C7F15)C branched forms, confirming none were present. Hence, a combination of structure-based filters and manual review confirmed 100% sensitivity of the PFOA SMILES Workflow (i.e., no False Negatives).

**FIGURE 3 |:**
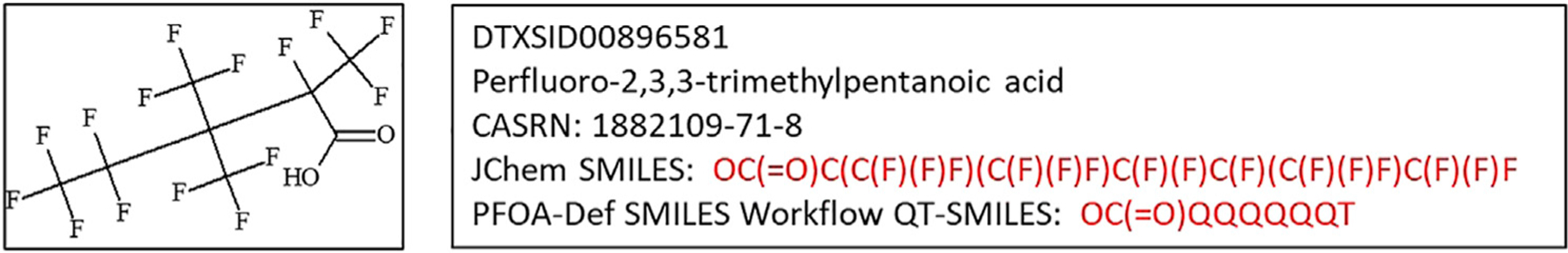
Sample structure for a branched isomer of PFOA with its chemical identifiers, including the JChem SMILES and its transformed QT-SMILES resulting from the PFOA SMILES Workflow.

Our approach used a series of SMILES transformations to convert branched forms of a perfluoroalkyl chain of specified formula, e.g., (C7F15)C, to the corresponding linear form. A sample of an original and transformed QT-SMILES is shown in Figure 3 for a representative branched isomer of PFOA. As mentioned previously, our approach largely relied upon the observation that perfluoroalkyl chains within JChem SMILES were contiguous, i.e., not split by functional groups as in the last PFOA SMILES example (#4) of the previous section. Note that after the PFOA SMILES Workflow In-rule transformations were applied to the JChem SMILES, PFOA and each of its 38 branched isomers, as well as corresponding salts and ionic forms, were converted to the same QT-SMILES as shown in this figure. In addition, all cases of linear forms of the (C7F15)C moiety embedded in larger chemicals were detected, without exception, as confirmed by the linear form substructure search.

**FIGURE 4 |:**
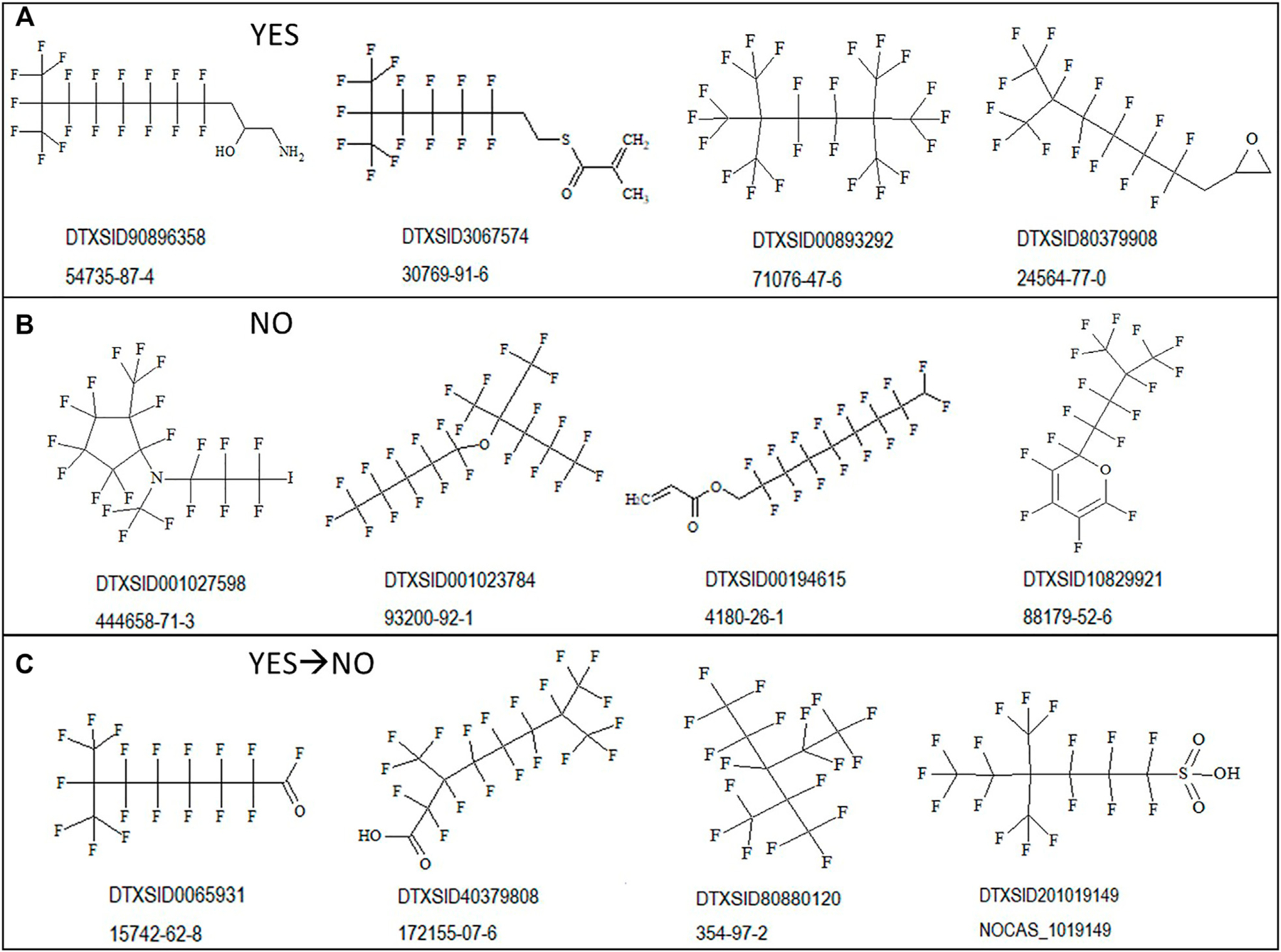
Sample structures for possible outcomes when applying the PFOA Workflow shown with the associated DTXSID and CASRN: **(A)** structures falling within the SC PFOA listing (YES); **(B)** structures not falling within the SC PFOA listing, with molecular formula containing C8F15 (NO); and **(C)** structures satisfying the In-rule but whose result is negated by an Out-rule exception.

To illustrate the diversity of structures correctly discerned by the PFOA SMILES Workflow, examples of branched and unbranched structures found within the various outcome bins are shown in Figure 4 below. Figure 4A shows “YES” structures falling within the SC PFOA listing (i.e., In-rule + Out-rule), Figure 4B shows “NO” structures not falling within the listing (containing at minimum the C8F15 formula), and Figure 4C shows YES → NO structures satisfying the In-rule but with the result negated by an Out-rule exception.

**FIGURE 5 |:**
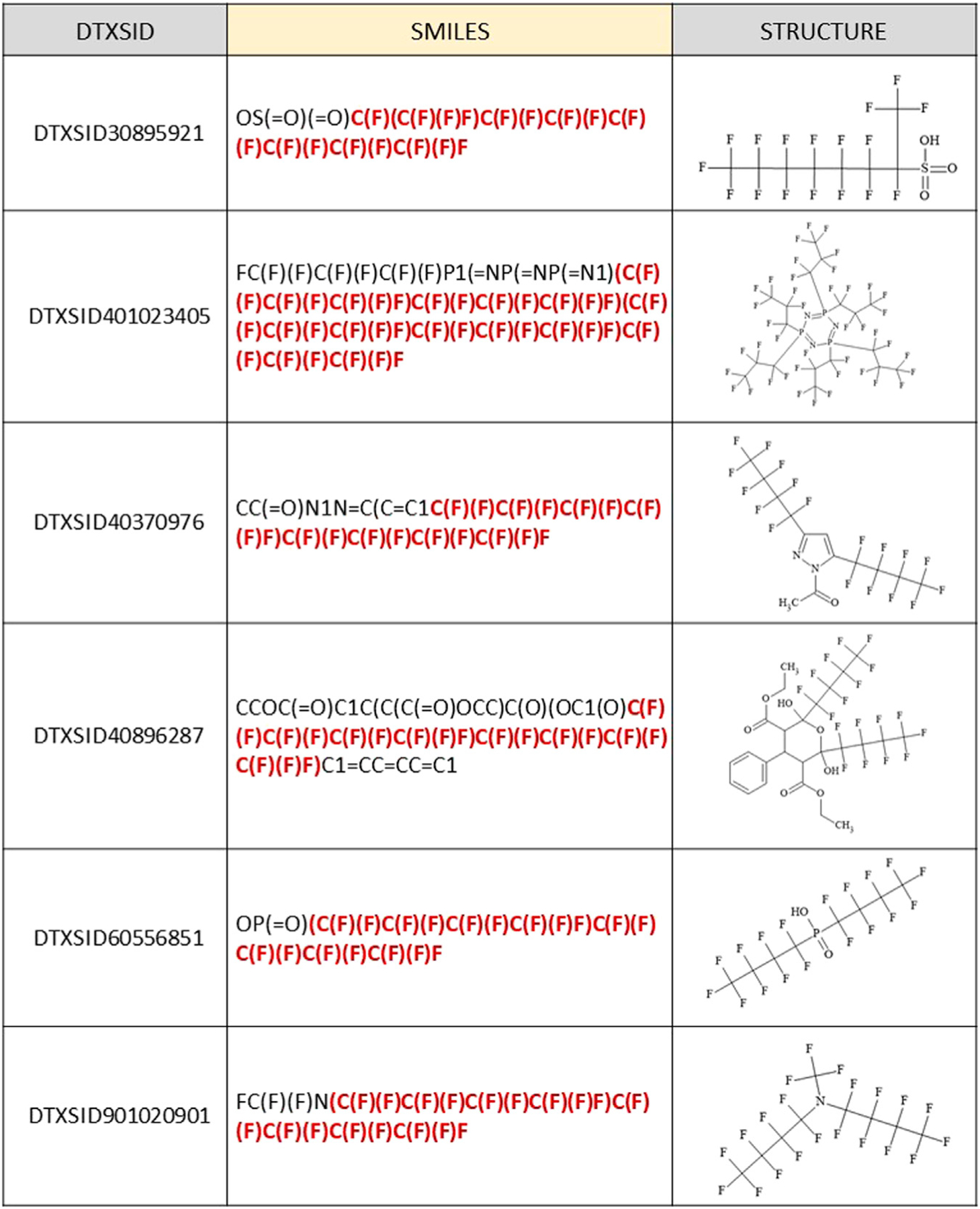
Examples of JChem SMILES and corresponding structures that were falsely predicted to fall within the SC PFOA listing (5 out of 35 total shown) due to contiguous representation of multiple perfluoro chains shorter than C7F15, with the portion of the SMILES that was misinterpreted highlighted in red.

As previously mentioned, there were only 36 false positives (out of more than 2000 total) where a (C7F15)C moiety was incorrectly predicted to be present as a branched form in the PFASSTRUCT+ set. One of these satisfied an Out-rule, so was converted to a “NO.” The majority of the remaining false positives (34/35) contained multiple smaller perfluoro chains written contiguously in the JChem SMILES, which were misinterpreted by our algorithm as a contiguous single chain. Additionally, two chemicals included a non-C functional group attachment before the (C7F15) chain completion. Five examples of these incorrectly classified structures are shown in Figure 5. The topmost structure has a C7F14 chain attached to the terminal C; hence, it is one fluorine short of the C7F15 condition. The remaining 4 structures are representative of the 34 misidentified structures containing multiple smaller perfluoro chains, with the portion of the SMILES misinterpreted as contiguous by our algorithm highlighted in red.

**FIGURE 6 |:**
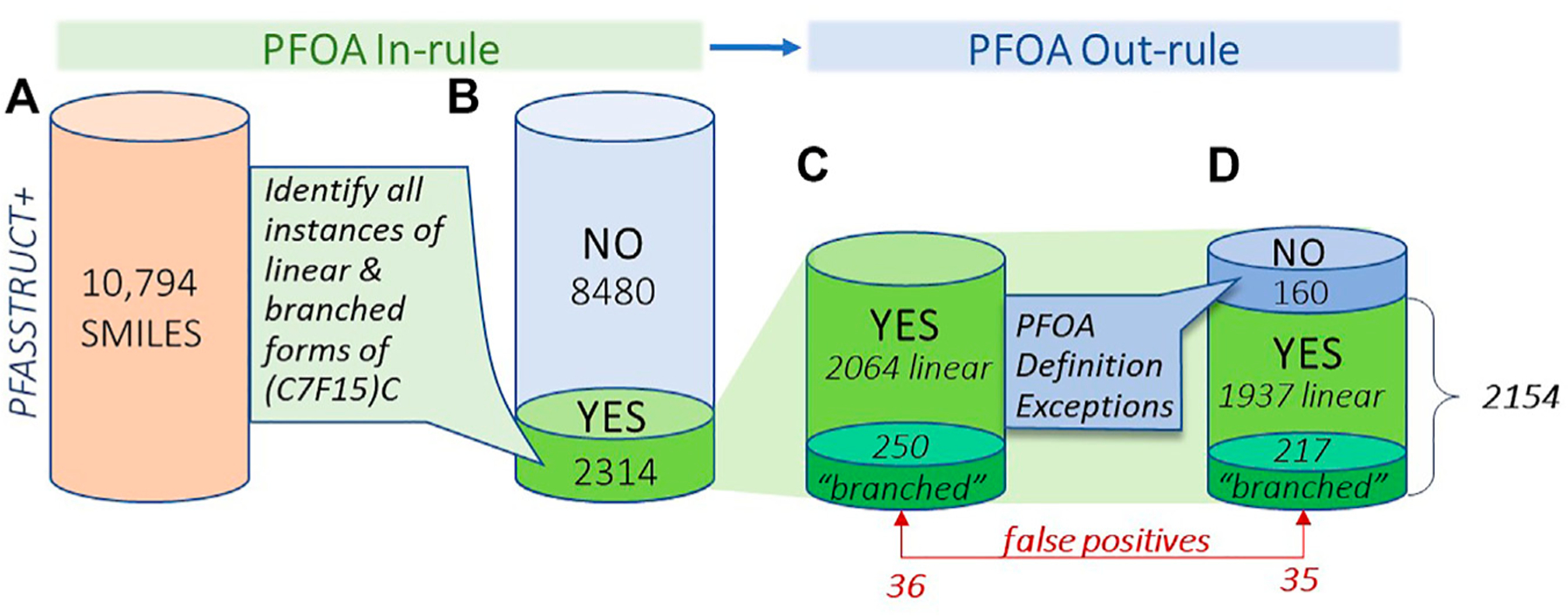
Visual summary of the results of application of the PFOA In-rule and PFOA Out-rule to the PFASSTRUCT+ SMILES set, showing numbers of structures falling into each bin, moving from left to right; **(A)** initial set of PFASSTRUCT+ **(B)** results after application of the In-rule (YES,NO); **(C)** structures satisfying the In-rule (YES), showing totals in the linear and branched subsets **(D)** totals after application of the Out-rule exceptions, with adjusted totals of linear and branched YES results.

Figure 6 provides a visual summary of the results of the PFOA SMILES Workflow, broken into its two major components: the PFOA In-rule, which determines all possible candidates containing branched or linear forms of the moiety (C7F15)C; and the PFOA Out-rule, which can convert In-rule YES candidates to NO, thus removing them from further consideration. We have further broken down the proportion of chemicals that initially satisfied the PFOA In-rule into branched and linear subsets, both before and after applying the Out-rule exceptions. Note that the transformed QT-SMILES were the means by which branched isomers were discerned in both the In-rule and Out-rule portions of the workflow, such that branched forms of the Out-rule exceptions were also detected (e.g., in the case of PFOS and its branched isomers). Indeed, 33 out of 160 total structures, whose initial YES assignments were reversed to NO on application of the PFOA Out-rule, were confirmed to be branched.

## DISCUSSION

The results in Figure 6 provide a view of the landscape of PFAS chemicals in commerce or detected in the environment that highlights the proportion of branched and linear forms of PFOA or substructures believed capable of degrading to PFOA. It is a surprisingly large proportion - nearly 20% (2154/10,794) of the total are predicted to fall within the SC PFOA listing. Hence, in addition to the 299 “YES” compounds in the 2021 PFOA Indicative List, we have added 1855 new “YES” compounds that fall within the SC PFOA listing. Furthermore, insight has been gained into the proportion of branched forms relative to linear forms of perfluoroheptyl groups in this large collection of PFAS structures, i.e., approximately 10% of the total (217/2154). This estimated percentage of unique branched isomers of the (C7F15)C moiety is almost certainly an underestimate of the true environmental total given the propensity of the PFAS community to represent PFAS chemicals in linear forms only (e.g., PFOA and PFOS), as well as the difficulty of differentiating linear from branched isomers with standard analytical methods, such as liquid chromotography mass spectroscopy (LCMS) typically applied to environmental samples. Hence, the proportion of unique branched isomers in commerce and the environment is most likely much higher than represented in the PFASSTRUCT+ file. In addition, the number of unique branched isomers for a particular linear perfluoro chain length increases by more than a factor of two for each additional CF2 carbon added, i.e., there are 38 unique branched isomers of PFOA, 89 for perfluorooctyl (C8F15) chains, and 211 for perfluorononyl (C9F19) chains.

Although the results of the present PFOA SMILES Workflow demonstrate excellent accuracy and recall in predicting whether a structure falls within the SC PFOA listing, with 100% sensitivity of true positives in the large PFASSTRUCT+ file, the approach we’ve taken is largely heuristic and retrospective. We use the term heuristic because our approach is only partially based on first principles, to the extent that the JChem SMILES consistently represent the (C7F15)-C moiety and the exception criteria. Furthermore, the derivation and success of our PFOA SMILES Workflow is dependent on use of JChem canonical SMILES, where the canonicalization algorithm and rules are unknown and can only be inferred from examples. The degree of this application-specific dependence was demonstrated by processing an alternate set of SMILES for the PFASSTRUCT+ file generated by ACD/Labs Spectrus software using our PFOA_SMILES_macro_v1. xlsm file. Direct comparison of the two sets of SMILES yielded fewer than 10% exact matches. As shown earlier for PFOA, different nodes (i.e., atoms) chosen for starting transversal along the chain and different paths chosen at branch nodes when constructing SMILES strings are responsible for these discrepancies. As a result, when we compared the PFOA In-rule and Out-rule final results, only 35% of the JChem SMILES YES predictions were reproduced by the ACD/Labs SMILES and there were more than 1100 false positive YES’s predicted by the latter (results not shown).

Despite the acknowledged limitations of the current approach, we have succeeded in one of our main objectives, which was to generate a much larger indicative list of compounds to represent the SC PFOA listing for the user community. The PFASSTRUCT+ file, with 10,794 structures, is 32 times larger than the 2021 PFOA Indicative List of 335 structures, with 1855 new compounds added to the previous 299 structures from the 2021 PFOA Indicative List labeled as falling within the SC PFOA listing. In addition, implementation of the PFOA SMILES Workflow within an MS Excel macro file provides the PFAS community with a user-friendly means for non-experts to evaluate whether a new PFAS structure is likely to fall within the SC PFOA listing. It is certainly the case that new and novel PFAS structures could be designed whose JChem SMILES would be falsely predicted by our rules, and the extension of the present approach to recognition of all possible branched members of chains longer than C7F15 would have to be confirmed. In addition, our approach cannot presently be applied to polymers and mixtures unless they are mapped to structural subcomponents (such as is possible with Markush representations and Markush enumeration-enabled software). However, demonstration of the accuracy of our PFOA SMILES Workflow in processing more than 10,700 structurally diverse PFASs provides confidence that the approach can serve as a valuable structure-based screening tool.

## CONCLUSION

The present study was prompted by a desire to translate the Stockholm Convention PFOA listing into a set of clear structure-based rules that could be used to evaluate large, and growing lists of PFAS structures to determine whether they fall within the parameters of the definition. The SC PFOA listing (Stockholm Convention POPRC 2019) appears deceptively simple but is in fact quite cheminformatically complex and challenging to apply. Language in the document such as “PFOA, including any of its branched isomers; its salts; and PFOA-related compounds … having a linear or branched perfluoroheptyl group with the moiety (C7F15)C as one of the structural elements” requires not only structure desalting, but the ability to recognize all possible linear and branched forms of the (C7F15)C moiety. The formula itself is somewhat restrictive in that it requires at least one CF3 group to achieve the F15 count, meaning the perfluoroheptyl group must be terminal to the structure, i.e., unbound on at least one end. Similarly, language in the document that defines exceptions, e.g., “phosphonic acids (including their salts, esters, halides and anhydrides) with ≥ 8 perfluorinated,” requires the ability to computationally recognize several types of functional groups, as well as their contiguous association with branched and linear perfluoro chains of specified lengths. Publishing the 2021 PFOA Indicative List of 393 PFAS examples falling in and out of the SC PFOA listing is helpful but is far too limited in its coverage of the current PFAS structure landscape, as well as is lacking examples for several of the Out-rule exclusion conditions. In addition, some types of structures not represented in the 2021 PFOA Indicative List and not strictly covered under the SC PFOA listing, such as those containing non-aromatic perfluoro rings, may not have been anticipated in early drafting of the definition.

Although efforts by others have attempted to classify PFAS chemicals by cheminformatics means with some degree of success (Sha et al., 2019; Su and Rajan 2021), there is no publicly available chemistry application, nor any commercial structure-handling software that we are aware of, that allows a user to identify all branched isomers of (C7F15)C within a diverse set of structures. The results presented here indicate that a simple substructure search for only linear forms of (C7F15)C would miss more than 10% of the total PFASs falling within the SC PFOA listing in the PFASSTRUCT+ file. Development of a structure-based approach capable of detecting all branched isomers of the (C7F15)C moiety with 100% accuracy is feasible but would likely require a separate substructure search for each of the 38 individual branched isomer fragments. In addition, such an approach would rely upon specialized cheminformatics software such that a web-based application delivered in a user-friendly way would be needed to encourage use by the broader PFAS community. Our decision to base our approach on SMILES was designed to circumvent these difficulties, use off-the-shelf, publicly available SMILES (through the Dashboard) and a spreadsheet application widely available to the public, researchers, and regulators world-wide (i.e., MS Excel).

Finally, a larger objective of the present work was to promote the use of unambiguous structure-based tools and definitions to support clear communication and regulatory action within the PFAS community. Due to the difficulties of computationally translating such a broadly stated, chemically complex definition to the processing of a large list of PFAS chemicals, there is a potential for misinterpretation and misapplication of the SC PFOA listing by the broader community. This work has also shed light on some of the limitations of structure-based approaches as applied to the current needs of the PFAS community. It is hoped that the present work addresses both an immediate need of the PFAS community, i.e., to provide a much larger indicative list of PFOA and related substances falling under the SC PFOA listing, as well as spurs the cheminformatics community to tackle the challenge of characterizing PFAS branching and associated chemical concepts of importance to the regulatory concerns relevant to PFAS chemicals.

## Supplementary Material

Supplement1

Supplement2

## Figures and Tables

**TABLE 1 |:** Confusion matrix relating the performance of the PFOA SMILES Workflow, separating In-rule and In-rule + Out-rule, when applied to the 10,794 SMILES contained in the PFASSTRUCT+ file where TP = #True Positives, FP = #False Positives, TN = #True Negatives, FN = #False Negatives, Accuracy=(TP + TN)/Total, Sensitivity = TP/(TP + FN).

PFOA SMILES workflow	Total	TP	FP	TN	FN	Accuracy (%)	Sensitivity (%)
UNEP POPRC PFOA Indicative List							
PFOA In-rule	335	299	22	14	0	93	100
PFOA In-rule + Out-rule	335	299	0	36	0	100	100
All-PFAS-Structures							
PFOA In-rule	10,785	2277	36	8,472	0	99.7	100
PFOA In-rule + Out-rule	10,785	2,105	35	8,645	0	99.7	100

## Data Availability

The original contributions presented in the study are included in the article/Supplementary Material, further inquiries can be directed to the corresponding author.

## Abbreviations:

COP: Conference of the Parties
Dashboard: CompTox Chemicals Dashboard
PFAS: per- and polyfluorinated alkyl substance
PFOA: perfluorooctanoic acid
PFOS: perfluorooctanesulfonic acid
OECD: Organisation for Cooperation and Economic Development
POP: Persistent Organic Pollutants
SC: Stockholm Convention
SMILES: Simplified Molecular-Input Line-Entry System
EPA: U.S. Environmental Protection Agency
